# Whole Genome Sequence Typing to Investigate the *Apophysomyces* Outbreak following a Tornado in Joplin, Missouri, 2011

**DOI:** 10.1371/journal.pone.0049989

**Published:** 2012-11-27

**Authors:** Kizee A. Etienne, John Gillece, Remy Hilsabeck, Jim M. Schupp, Rebecca Colman, Shawn R. Lockhart, Lalitha Gade, Elizabeth H. Thompson, Deanna A. Sutton, Robyn Neblett-Fanfair, Benjamin J. Park, George Turabelidze, Paul Keim, Mary E. Brandt, Eszter Deak, David M. Engelthaler

**Affiliations:** 1 Centers for Disease Control and Prevention, Atlanta, Georgia, United States of America; 2 Translational Genomics Research Institute, Flagstaff, Arizona, United States of America; 3 Department of Pathology, University of Texas Health Science Center, San Antonio, Texas, United States of America; 4 Missouri Department of Health, Saint Louis, Missouri, United States of America; Georgia Institute of Technology, United States of America

## Abstract

Case reports of *Apophysomyces* spp. in immunocompetent hosts have been a result of traumatic deep implantation of *Apophysomyces* spp. spore-contaminated soil or debris. On May 22, 2011 a tornado occurred in Joplin, MO, leaving 13 tornado victims with *Apophysomyces trapeziformis* infections as a result of lacerations from airborne material. We used whole genome sequence typing (WGST) for high-resolution phylogenetic SNP analysis of 17 outbreak *Apophysomyces* isolates and five additional temporally and spatially diverse *Apophysomyces* control isolates (three *A. trapeziformis* and two *A. variabilis* isolates). Whole genome SNP phylogenetic analysis revealed three clusters of genotypically related or identical *A. trapeziformis* isolates and multiple distinct isolates among the Joplin group; this indicated multiple genotypes from a single or multiple sources. Though no linkage between genotype and location of exposure was observed, WGST analysis determined that the Joplin isolates were more closely related to each other than to the control isolates, suggesting local population structure. Additionally, species delineation based on WGST demonstrated the need to reassess currently accepted taxonomic classifications of phylogenetic species within the genus *Apophysomyces*.

## Introduction

The fungus *Apophysomyces* is a member of the order Mucorales associated with invasive mucormycosis [1], [2], [3], [4], [5], [6], [7], [8], [9], [10], [11], [12]. In contrast to other members of the Mucorales, human infections with *Apophysomyces* spp. have been identified mainly in immunocompetent hosts as a result of traumatic inoculation of infective spores [8], [13], which can be isolated from soil, decaying vegetation, detritus, and other organic substrates rich in simple carbohydrates [2]. Of 74 reported cases of *Apophysomyces* spp. infections identified prior to this outbreak, the majority have occurred as a result of motor vehicle, contamination of burn wounds, and natural disasters such as tsunamis [14].

Recent taxonomic revision of *Apophysomyces*, based on the use of ribosomal DNA and protein coding regions [15], determined that the genus may be comprised of a complex of four species: *A. elegans*, *A. ossiformis*, *A. trapeziformis*, and *A. variabilis*. In recent studies, molecular typing of presumed isolates of *A. elegans* using microsatellites and AFLP revealed very little genetic diversity [2], [16], however, advanced molecular typing methods with improved phylogenetic and resolving power have yet to be performed. Such molecular studies are needed to understand the distribution of *Apophysomyces* spp. within the taxonomic complex and provide insight into the genetic differences among the species that may reveal varying environmental and clinical phenotypes.

In May 2011, following an F5 category tornado in Joplin, MO, 13 cases of *Apophysomyces* infections (typed as *A. trapeziformis*) were observed, but no specific environmental source or common exposure was identified [17], [18]. We used whole genome sequence typing (WGST) and a whole genome SNP profile analysis tool to interrogate the relatedness of the *A. trapeziformis* isolates from this outbreak. Whole genome sequence typing has emerged as a robust molecular epidemiology and phylogenetic methodology, especially with pathogens such as *Apophysomyces*, where the availability and reliability of molecular tools to understand genetic diversity are lacking. In addition, WGST is increasingly being used to understand the genetic diversity of medically important fungi implicated in clusters of disease [19], [20]. We describe the use of WGST to investigate the genetic diversity of *A. trapeziformis* isolates from 11 cases infected during the Joplin, MO tornado.

## Methods

### Isolates

Seventeen isolates from eleven Joplin cases were processed by the Mycotic Diseases Branch (MDB), CDC, Atlanta, GA for ribosomal DNA sequencing and WGST analysis. Isolates Apo 095, 097, 098, 099, 884 and 846 were single isolates from six Joplin tornado victims (Table 1). Isolates Apo 100, 101, 105, and 106 originated from different body sites of the same patient, as did Apo 110/845, Apo 107/108, and Apo 813/814. Isolate Apo 793572 was received from the Fungus Testing Laboratory, University of Texas Health Science Center (UTHSC) at San Antonio, San Antonio, TX. In addition, five non-outbreak *Apophysomyces* isolates (referred to as control isolates) were included for analysis. Four of these isolates [Apo 7450, 7449, 7451, 7759] were also provided by the UTHSC Fungus Testing Laboratory, and were previously described as either *A. trapeziformis* or *A. variabilis*, based on multilocus DNA sequence analysis [15]. The fifth isolate [Apo 7452] was from a patient who developed renal mucormycosis following transplantation of a kidney from a donor who had been involved in a near-drowning event during a motor vehicle accident. This isolate, previously described [21], was originally identified as *A. elegans* based on morphologic characteristics and analysis of ITS, and 28S LSU rDNA sequences. All isolates have been stored in the MDB isolate collection as listed above, respectively B9336, B9235, B9236, B9237, B9340, B9341, B9328, B9329, B9330, B9331, B9334, B9337, B9332, B9333, B9335, B9338. Additionally, these isolates have been banked in the USDA Agricultural Research Service (ARS) Culture Collection.

**Table 1:** List of strains used in study, by isolation site, species and geographic location, along with sequencing coverage results.

Strain ID	Cluster ID	Type	Body Site	Species ID	Location	# Bases >10× Coverage (% Ref Genome)
Apo-813	C1-A*	Clinical	L.flank tissue	A. trapeziformis	Joplin, MO	34,124,335 (99.3%)
Apo-814	C1-A*	Clinical	L.flank tissue	A. trapeziformis	Joplin, MO	34,072,889 (99.2%)
Apo-097	C1-B	Clinical	scalp	A. trapeziformis	Joplin, MO	34,328,236 (99.9%)
Apo-884	D	Clinical	tissue sacral wound	A. trapeziformis	Joplin, MO	34,066,466 (99.2%)
Apo-793572	E	Clinical	isolate	A. trapeziformis	Joplin, MO	33,596,472 (97.8%)
Apo-846	C2-F	Clinical	Scalp wound	A. trapeziformis	Joplin, MO	34,009,289 (99.0%)
Apo-100	C2-G*	Clinical	temporal tissue	A. trapeziformis	Joplin, MO	34,075,887 (99.2%)
Apo-101	C2-G*	Clinical	scalp	A. trapeziformis	Joplin, MO	34,113,366 (99.3%)
Apo-105	C2-G*	Clinical	Left pariental region	A. trapeziformis	Joplin, MO	34,095,842 (99.3%)
Apo-106	C2-G*	Clinical	scalp	A. trapeziformis	Joplin, MO	34,073,965 (99.2%)
Apo-098	H	Clinical	L. flank	A. trapeziformis	Joplin, MO	33,614,918 (97.9%)
Apo-110	C3-I*	Clinical	Cheek	A. trapeziformis	Joplin, MO	33,801,232 (98.4%)
Apo-099	C3-J	Clinical	R. leg abscess	A. trapeziformis	Joplin, MO	33,802,988 (98.4%)
Apo-095	C3-K	Clinical	Leg muscle	A. trapeziformis	Joplin, MO	33,933,736 (98.8%)
Apo-845	C3-I*	Clinical	skin site/Left cheek	A. trapeziformis	Joplin, MO	34,118,912 (99.3%)
Apo-107	L-1	Clinical	L.hip drainage	A. trapeziformis	Joplin, MO	34,015,778 (99.0%)
Apo-108	L-2	Clinical	L.hip tissue	A. trapeziformis	Joplin, MO	33,774,176 (98.3%)
Apo-7450	NA	Clinical	Abdominal tissue	A. trapeziformis	Phoenix, AZ	34,065,390 (99.2%)
Apo-7449	NA	Dolphin	N/A	A. variabilis	Bahamas	26,658,620 (77.6%)
Apo-7451	NA	Clinical	Skin Biopsy	A. trapeziformis	Colorado	33,701,402 (98.1%)
Apo-7452	NA	Clinical	Organ transplant	A. trapeziformis	North Carolina	33,661,024 (98.0%)
Apo-7759	NA	Clinical	N/A	A. variabilis	Netherlands	32,322,512 (94.1%)

NA = Not Applicable; N/A = Not Available; C = Cluster;

* indicates multiple isolates from the same patient.

All isolates were plated on Sabouraud Dextrose Agar (SDA) with gentamicin and chloramphenicol to ensure purity. Isolates were inoculated into tap water supplemented with 0.1% yeast extract to induce sporangiospore production [22]. Sporangiospores were harvested by transferring the tap water cultures to 50 ml conical tubes and centrifuging at maximum speed (14,000 rpm) for 3 minutes. Supernatants were discarded and resultant pellets were re-suspended in 1 ml of sterile distilled water. Concentrations of suspended sporangiospore stocks were determined by counting the sporangiospores using a hemocytometer chamber at 400× magnification and stocks were diluted in sterile distilled water to a final concentration of 1 spore/10 µl. Ten microliters of each isolate was mixed with 500 µl of distilled water and plated on SDA plates for single spore cultures. After 24 hrs, plates were verified for growth of a single colony, presumed to be the progeny of one sporangiospore. Single spore plates were allowed to grow for an additional 72 hours, after which DNA was extracted.

DNA was isolated using the GeneRite kit (North Brunswick, NJ) according to the manufacturer's directions. DNA products were quantified using a Nanodrop (ThermoScientific, Asheville, NC) and stored at −20°C until further use.

### Species identification

DNA from all isolates was subjected to PCR amplification and sequence confirmation using the D1/D2 28S LSU rDNA region [15]. PCR amplification was performed in a 50 µl reaction mixture using PCR Master Mix according to the manufacturer's protocol (Roche Scientific, San Francisco, CA). The reaction conditions were: initial denaturation at 95°C for 5 min; 35 cycles of 95°C for 30 sec, 50°C for 45 sec, 72°C for 1 min; and a final elongation step at 72°C for 5 min.

### Whole Genome Sequencing

The DNA samples were prepared for multiplexed, paired-end sequencing on the Illumina GAIIx Genome Analyzer (Illumina, Inc, San Diego, CA), as previously described [20], [23], with minor modifications. For each isolate, 1–5 µg dsDNA in 200 µl was sheared in a 96-well plate with the SonicMAN™ (Matrical Bioscience, Spokane, WA) to a size range of 200–1000 base pairs with the majority of material at ca. 600 base pairs using the following parameters: Pre Chill - 0°C for 75 sec; Cycles - 20; Sonication - 10 sec; Power - 100%; Lid Chill - 0°C for 75 sec; Plate Chill - 0°C for 10 sec; Post Chill - 0°C for 75 sec. The sheared DNA was purified using the QIAquick PCR Purification kit (Qiagen,Valencia, CA). The enzymatic processing (end-repair, phosphorylation, A-tailing, and adaptor ligation) of the DNA followed the guidelines as described in the Illumina protocol (“Preparing Samples for Multiplexed Paired-End Sequencing”, Catalog #PE-930-1002). The enzymes for processing were obtained from New England Biolabs (Ipswich, MA) and the oligonucleotides and adaptors were obtained from Illumina. After ligation of the adaptors, the DNA was run on a 2% agarose gel for 2 hours, after which a gel slice containing 500–600 bp fragments of each DNA sample was isolated and purified using the QIAquick Gel Extraction kit (Qiagen, Valencia, CA). Individual libraries were quantified with qPCR on the ABI 7900HT (Life Technologies Corporation, Carlsbad, CA) in triplicate at two dilutions, 1∶1000 and 1∶2000, using the Kapa Library Quantification Kit (Kapa Biosystems, Woburn, MA). Based on the individual library concentrations, equimolar pools of no more than 4 indexed *Apophysomyces* libraries were prepared at a concentration of at least 5 nM using 10 mM Tris-HCl, pH 8.0+0.05% Tween 20 as the diluent. To ensure accurate loading onto the flowcell, the same quantification method was used to quantify the final pools. The pooled, paired-end libraries were sequenced on the Illumina GAIIx to a read length of 101 base pairs. The sequence data files have been deposited in the National Center for Biotechnology Information's Sequence Read Archive (SRA).

### Assembly

An *Apophysomyces* genome reference was assembled *de novo* from raw sequencing reads from isolate Apo 097 using Edena [24]. The final assembly contained 34,350,872 total bases in 2,616 contigs with an n50 of 30,896, and an average G/C content of 42% (Table 2). A self-alignment of the Apo 097 raw reads to the assembled contigs resulted in 47 false SNP calls (1 false SNP/731kbp) indicating a high quality assembly. These false SNP loci were subsequently removed from the WGST analysis.

**Table 2:** *De novo* assembled reference statistics for Strain Apo-097.

Reference	Raw Reads into Assembly	% Reads Assembled	# Bases in Assembled Genome	N50	N80	Total # Contigs	% G/C
Apo-097	37,136,469	80	34,350,872	30,896	13,316	2,616	41.9

### Alignment

Illumina WGS data sets were aligned against the *de novo* reference assembly (Isolate Apo 097) using the short-read alignment component of the Burrows-Wheeler Aligner (BWA) alignment tool [25]. Reads containing insertions or deletions, and those mapping to multiple locations in the reference were removed from the final alignments.

#### Identification of single nucleotide polymorphism (SNPs)

SNP detection was conducted using SolSNP (http://solsnp.sourceforge.net/), a publically available in-house developed tool. SNPs were excluded if they did not meet a minimum coverage of 10× and if the variant was present in less than 90% of the base calls for that position. Subsequently, regions found to be duplicated in the Apo 097 reference genome were identified using MUMmer version 3.22; SNPs within these repetitive regions were then removed. Loci that lacked ≥10× reference sequence coverage data for one or more isolates were also removed from the final analysis. The remaining orthologous SNPs (loci shared across all genomes) were placed in a matrix for phylogenetic analysis.

#### Phylogenetic analysis

Whole genome sequence typing (WGST)-based phylogenetic analyses were performed using the maximum parsimony algorithm, default parameters, in MEGA5 [26]. Where shown, 1000 generations were run for bootstrap analysis. Reference genome mapping statistics were determined using a custom script; read depth statistics were determined using the Genome Analysis Toolkit [27]. A Mantel test using GENALEX [28] was performed to determine relationship between genetic distance of the strains and the geographic distance of location of exposure, in order to determine any phylogeographic patterns.

## Results

### Species identification

Seventeen isolates from eleven cases were evaluated for analysis. Isolates were identified as *A. trapeziformis* based on 28S rDNA sequencing results. Among the five control isolates, three isolates were identified as *A. trapeziformis* and two isolates were identified as *A. variabilis*. All seventeen outbreak isolates and five control isolates were further analyzed using whole genome sequencing (WGS).

### Sequence analysis

Twenty-one of the twenty-twogenome sequences mapped to at least 98% of the *de novo* reference (with >10× coverage), with two genome sequences mapping to 77.6% and 94.1%, respectively (Table 2). Based on the total length of the combined reference genome contigs, the genome size is estimated to be between 31.9 and 32.1 Mb.

### Phylogenetic analysis

**Figure 1:**
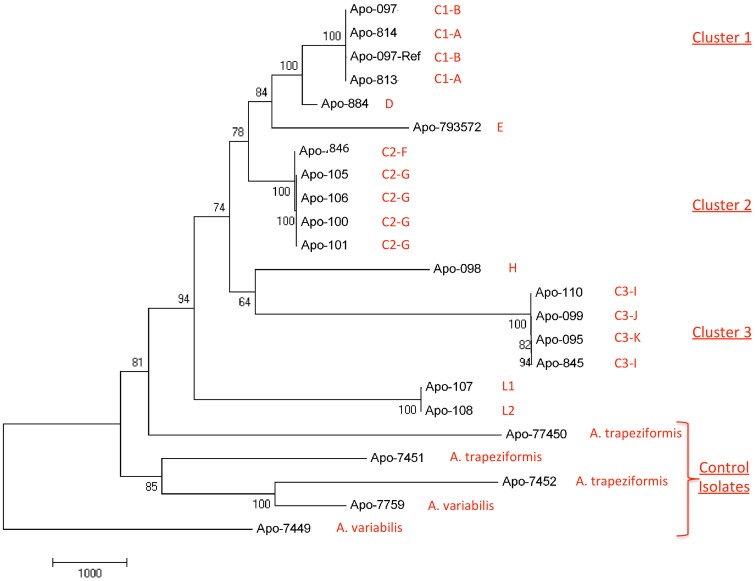
WGST phylogeny of outbreak and background Apophysomyces isolates. A single maximum parsimony tree was reconstructed using ∼28K SNPs from 22 whole genome sequences, resulting in a CI of 0.62. The tree was rooted with Apo-7449 (A. variabilis). The root was derived from an expanded phylogenetic analyses that included a distant outgroup, which enabled the identification of the most basal member of the samples in the dataset described in this figure (data not shown). Genomes are labeled with a Strain ID # (APO-XXXX) and a Cluster ID #-Letter (CX-A). Clusters of identical and nearly identical genome SNP profiles are also labeled. Branch lengths represent genetic distance based on the number of SNP differences; bar represents 1000 SNPs. Tree constructed using MEGA5.

**Figure 2:**
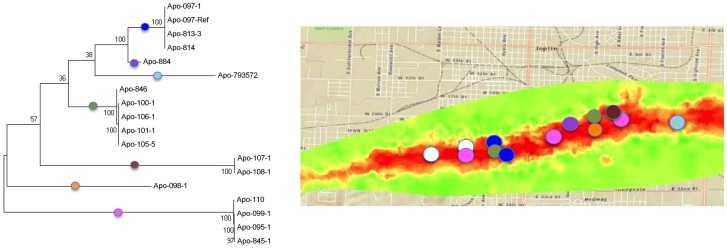
Locations of *Apophysomyces* exposure in Joplin, MO during tornado. Colored circles on the map represent exposure locations based on epidemiologic interviews [18]. Color represents severity of tornado based on structural damage, from red (catastrophic) to green (moderate) (Map credit: United States Army Corps of Engineers). N/A = isolate not available for analysis.

Phylogenetic analysis using WGST showed all Joplin outbreak isolates to be more closely related to each other than to the five *Apophysomyces* background isolates. Within the Joplin outbreak set, three clusters of identical or nearly identical SNP profiles (Cluster 1–3), as well as distinct isolates, were identified (Figure 1). The identified clusters within the Joplin isolate set (C1, C2 and C3) contained relatively few SNPs (n = 2, 27, and 15, respectively). These clusters contained isolates from multiple cases. Four cases (Apo 110/845, Apo 100/101/105/106, Apo 107/108, Apo 813/814) had multiple isolates from disparate body sites. In each case, the individual isolates were nearly genotypically indistinguishable and represented a single strain. The results of the Mantel test determined there was no association between geographic and genetic distance and therefore no discernable relationship between the location of exposure and the genotype of the strains (Figures 2 and S1).

The WGST phylogeny showed far more genetic distance among the control strains than among the Joplin outbreak isolates and it was unable to separate out these strains by species (Figure 1). In particular, one of the *A. trapeziformis* isolates (Apo 7452) in the background cluster appeared genetically closer to one of the *A. variabilis* isolates (Apo 7759) than to the Joplin *A. trapeziformis* isolates, although 28s rDNA sequencing suggests that they are the same species. A relatively low consistency index (CI) value of 0.62 indicates significant levels of homoplasy(i.e., similiarity in character states that arise from processes independent of inheritance from a common ancestor, such as convergent evolution, recombination, and lateral gene transfer), possibly due to high levels of recombination and lateral gene transfer, which can decrease the accuracy and clarity of phylogenetic reconstructions.

## Discussion

Though individual cases and outbreaks due to *Apophysomyces* spp. remain rare, these infections are very serious and often fatal. The Joplin, Missouri *Apophysomyces* outbreak is the largest documented cluster of this fungal genus. As *Apophysomyces* species are considered soil dwelling saprobes causing disease following traumatic inoculation, infections occurring after multiple trauma following an F5 tornado may not be unexpected. Joplin, MO and the surrounding region previously received increased rainfall in the weeks leading up to the May 2011 tornado [29]. Although only speculative, such conditions may have allowed for local blooms of distinct populations just prior to the tornado hitting the region. It is also possible that fungal spores were picked up from one or more sites along the western edge, where the tornado first touched down, allowing for multiple populations to be carried and dispersed along the remainder of the tornado's path, or genetically mixed populations may have been picked up at multiple sites along the path of the tornado. Follow-up investigations to date have not recovered *Apophysomyces* populations from the immediate or surrounding environment. Interestingly, we contacted state health departments in Alabama and Massachusetts following the tornadoes that occurred around the same time as in Joplin and there were no reported recoveries of *Apophysomyces* spp. from wound infections. The presence of distinct and diverse clonal clusters among the Joplin isolates suggests that *Apophysomyces* was previously well established in the Joplin, MO area. However, our lack of knowledge about the ecology and population genetics of this organism hampers the epidemiologic understanding of the nature of this outbreak.

The phylo-geographic analysis conducted here provides evidence of either a multi-focal source of *Apophysomyces* within a limited geographic area (e.g., several sites each with distinct *Apophysomyces* populations) or a single source with multiple clonal lineages (e.g., one site containing multiple *Apophysomyces* genotypes). The WGST-based phylogenetic analysis identified multiple clusters of cases infected with identical or near identical genotypes (Clusters 1–3). These clusters did not have any significant geographic linkage, although all isolates from the Joplin outbreak appear to be more closely related to one another than to the background isolates in this study. The lack of micro-scale geographic epidemiologic linkage is not surprising given the size of the tornado, the extent of the damage and the possibility of widespread distribution of multiple genotypes. Clusters of isolates having identical or near identical genomes provides evidence of local clonality, likely through asexual sporulation in the environment. In contrast, the separation of clusters by hundreds to thousands of SNPs is indicative of genetic diversity among strains, which can be caused by genetic . Although sexual reproduction has not been described for Apophysomyces, the low consistency index (CI = 0.62) suggests homoplasy and is further evidence of recombination and possible lateral gene transfer in this population. Overall, the presence of strains with identical as well as distinct genotypes is consistent with a working hypothesis of multiple distinct populations being aerosolized by the tornado from disrupted soil, water and other organic matter.

In the present study, WGST provides robust molecular epidemiologic analysis from a single relatively large outbreak. Previous typing techniques for *Apophysomyces* have included RFLP, AFLP, MLST and microsatellites [2], [15], [16], none of which are able to provide the level of resolution and phylogenetic understanding as whole genome SNP analysis. The whole genome-based phylogeny of *Apophysomyces* presented here suggests highly complex phylogenetic structure of this species complex, which has not been detected using other methods. The control strains used in this study were identified as *A. variabilis* and *A. trapeziformis* by recently described typing methodologies [15]; however they were not differentiated within the WGST tree, as would be expected for distinct species. Not surprisingly, there is significantly greater genetic diversity among the five non-Joplin control isolates (n = 17,000 SNPs), with little structure to indicate any degree of genetic relatedness among them. In addition, only one of the *A. trapeziformis* control isolates (Apo-7450) was more genetically related to the Joplin isolates than to the *A. variabilis* control isolates. While it is likely that multiple species of *Apophysomyces* do occur, the current designation of the four species may not hold true. For example, it is quite possible that the distinct Indian environmental strains typed as *A. elegans* by Alvarez [15] are a distinct species, as these have been previously shown to be quite genetically distinct from other *Apophysomyces* isolates [16]. Overall, our data underscores a need for reassessing the current understanding of the taxonomy and population genetics of this organism.

Limited sequence-based phylogenetic analyses such as MLST) can generate inconsistent results due to the confounding effects of recombination, lateral gene flow and variable evolutionary rates across the genome. However, the deleterious effects that the individual loci may have on the phylogeny can be mitigated by increasing the number of loci in the analysis [30]. By interrogating all common SNP loci between genomes, WGST allows for a highly accurate assessment of genetic and evolutionary relationships among isolates, providing both genotype (genetic linkage between isolates) and phylotype (phylogenetic placement within a given population). This, in turn, provides both empirical evidence of relatedness for epidemiologic purposes and places strains within an overall population structure. Whole genome sequencing also provides robust and comprehensive data for use in further comparative genomic explorations of the extraordinary virulence/host susceptibility, particularly in immunocompetent hosts; antifungal resistance properties; and strain to strain genomic variations that may govern the aforementioned factors.Additionally, WGS data can be compared among the various Mucorales genera and with non-Mucorales genera to allow further insight into novel mechanisms of virulence and potential targets for antifungal drugs in this group of human fungal pathogens. WGST has been used in other fungal and bacterial disease investigations [19], [20], [23], [31]. For this study, we developed the first population structure for *Apophysomyces* based on whole genome analysis, and provide an initial genomic view of this fungus in a natural setting.

The work presented here indicates a need to add many new whole genome sequences of diverse *Apophysomyces* strains to the existing phylogeny to further elucidate possible species separation within the genus and clarify the phylogenetic relationships within and among the member species. The paucity of isolates available in culture collections and the difficulty in maintaining viability, however, may hamper this pursuit. There is also a continued need to explore the natural ecology and epidemiology of *Apophysomyces*, particularly with an understanding where risks of outbreaks occur following similar large-scale community exposures.

## Supporting Information

Figure S1 **Results from Mantel test showing no relationship between genetic and geographic distance of isolates (Rxy = −0.088; p = 0.33).**
(TIF)
