# Supplementary figures and images for: Whole Genome Sequence Typing to Investigate the Apophysomyces Outbreak following a Tornado in Joplin, Missouri, 2011
Source: PLoS One. 2012 Nov 27;7(11):e49989. doi: 10.1371/journal.pone.0049989 (PMC3507928; doi:10.1371/journal.pone.0049989)

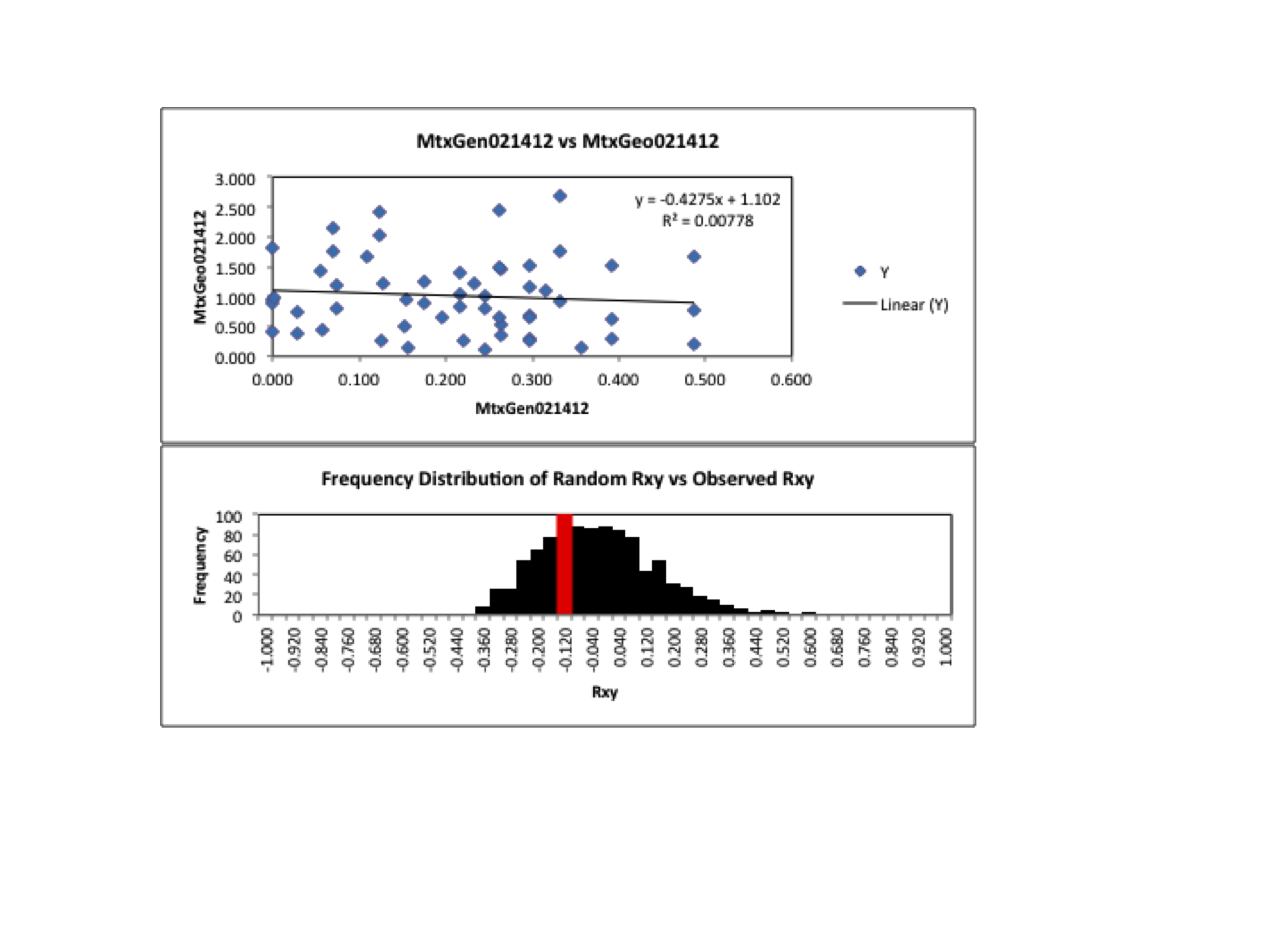

Supplement: Figure S1 — Results from Mantel test showing no relationship between genetic and geographic distance of isolates (Rxy = −0.088; p = 0.33). (TIF) [file pone.0049989.s001.tif]
